# Hospitalizations for bronchiolitis among infants before and after the SARS-CoV-2 pandemic: an area-based study of the Emilia-Romagna Region, Italy

**DOI:** 10.1186/s13052-025-01871-6

**Published:** 2025-02-07

**Authors:** Elisa Ballardini, Marco Manfrini, Silvia Fattori, Elena Pellacani, Branislava Ćosić, Giancarlo Gargano, Alberto Berardi

**Affiliations:** 1 Neonatal Intensive Care Unit, Dept. of Medical Sciences, University Hospital of Ferrara, IMER Registry (Emilia Romagna Registry of Birth Defects), University of Ferrara, Ferrara, Italy; 2 IMER Registry (Emilia Romagna Registry of Birth Defects), University Hospital of Ferrara, Ferrara, Italy; 3 Pediatric Postgraduate School, University of Ferrara, Ferrara, Italy; 4 Pediatric Postgraduate School, University of Modena and Reggio Emilia, Modena, Italy; 5 Neonatal Intensive Care Unit, Azienda Unità Sanitaria Locale-IRCCS, Reggio Emilia, Italy; 6 Neonatal Intensive Care Unit, Policlinico University Hospital, Modena, Italy

**Keywords:** Bronchiolitis, Respiratory syncytial virus, SARS-CoV-2, Infants, Epidemiology, Hospitalization

## Abstract

**Background:**

Bronchiolitis is the most frequent lower respiratory tract infection and a leading cause of hospitalization in infants. Our aim was to assess the incidence and characteristics of bronchiolitis requiring hospital admission in an Italian region before and after the SARS-CoV-2 pandemic.

**Methods:**

This area-based retrospective study analyses 4,396 hospital discharge records (HDR) of children under 1 year of age admitted with a diagnosis of bronchiolitis (ICD9-CM codes 466.11 and 466.19), in Emilia-Romagna (Italy) from January1st, 2018 to December 31th, 2021. Weighted t-testing and Z-testing was carried out.

**Results:**

in the study period, 2–4% of infants were admitted for bronchiolitis (10% of all admissions under 1 year) and 59% of them were aged less than 90 days. After a significant decrease in 2020, bronchiolitis resurged in 2021, and Respiratory Syncytial Virus (RSV) cases reached 82%. RSV cases were more likely to undergo non-invasive ventilation (NIV), oxygen supplementation and to receive i.v. (intravenous) infusions. There was an overall increasing trend in NIV and oxygen supplementation, and a decreasing trend in chest X-rays.

**Conclusions:**

This area-based study shows reduced hospital admissions due to bronchiolitis during the SARS-CoV-2 pandemic and a resurgence of RSV infection after the easing of preventive measures. We also provide information on length of stay and need for hospital treatments. These area-based information will be helpful in assessing the impact of future universal prevention measures.

## Background

Bronchiolitis is the most common cause of lower respiratory tract infection during the first year of life and a leading cause of pediatric hospitalization and death. The diagnosis is typically clinical, and the main symptom is rhinorrhea which progresses into respiratory distress associated with cough. The peak of severity occurs between the third and the fifth day after onset, with a full recovery within 2–3 weeks [1].

Bronchiolitis accounts for up to 15–17% of all hospitalizations in children younger than 2 years, but the incidence of bronchiolitis may be higher in children younger than 6 months of age. Respiratory syncytial virus (RSV) is the main cause of bronchiolitis [2].

RSV is an RNA virus that typically spreads in Italy from mid-October/November to March/April, with peak incidence occurring in January/February [3]. Globally, RSV is estimated to cause 34 million infections per year, 3 million of which require hospitalization. It is the leading cause of death from respiratory infection in children under 1 year of age [4, 5]. By the age of 2, all children are considered to have been infected by RSV, which is responsible for both upper and lower respiratory tract infection (LRTI) and is the leading cause of LRTI in children < 1 year of age [2].

Several underlying conditions (such as prematurity under 35 weeks of gestation, congenital heart diseases, bronchopulmonary dysplasia, cystic fibrosis, immunodeficiencies and others) increase the risk of severe infection, although recent evidence suggests a relevant rate of term and healthy infants requiring hospitalization [6, 7].

Several measures need to be improved to prevent infection and reduce the burden of hospital admissions, costs and health care management [8, 9].

The epidemiology of RSV has dramatically changed in recent years, due to SARS-CoV-2 pandemic related restrictions (such as social distancing, hand hygiene measures and the use of face masks) [2]. A substantial reduction in RSV hospitalizations, following a gradual lifting of restrictions, as well as the presence of the virus outside its typical seasonality, has been observed worldwide [10–12]. Decreased RSV circulation led to a relevant number of RSV-naïve individuals and a subsequent remarkable increase in the number of RSV-infected individuals [12–14]. Mathematical models predict that an increase in the susceptible population is an important risk for more severe epidemics in years to come [15].

Bronchiolitis (particularly RSV-related admissions) have a significant impact on the immediate clinical and economic burden, and long-term consequences, including impaired lung function, recurrent wheezing, and asthma [2, 6, 16]. Therefore, it is crucial to predict the peak incidence of RSV in order to immunize the most vulnerable population in a timely manner. Palivizumab, a specific monoclonal antibody, has been the only drug approved for preventing RSV infection. More recently, the monoclonal antibody Nirsevimab has been approved by the European Medicines Agency (EMA) for the protection of babies and toddlers, leading to a need for new preventive strategies [17, 18].

The circulation of RSV is not uniformly distributed throughout the world since each geographical area has its own variability in terms of incidence [2]. It is therefore crucial to have high level area-based data to manage hospital burdens and to evaluate prevention strategy efficacy [9]. However, Italian area-based data is limited to one study prior to the SARS-CoV-2 pandemic [19].

The aim of this retrospective, area-based study is to assess both incidence and characteristics of severe bronchiolitis requiring hospital admission between 2018 and 2021 in the Emilia Romagna region. This information could prove very useful in establishing the impact of future universal prevention measures.

## Methods

This is a retrospective, area-based study analyzing hospital discharge records (HDRs) of children under 1 year of age, admitted to the Emilia Romagna Regional Hospitals from January 1st 2018 to December 31st 2021 with a diagnosis of bronchiolitis, that does not take into account the infant’s residence. Cases have been identified by using the ICD9-CM coding system (International Classification of Disease, 9th Revision, Clinical Modification), which is currently applied in Italy.

All admission regarding infants in their first year of age with a diagnostic code 466.11 (RSV-related bronchiolitis) or 466.19 (non RSV-related bronchiolitis), both as primary or secondary diagnosis, were included. Children older than 365 days of life were excluded. HDR data is provided (as aggregate data) by the Regional Health Service of Emilia-Romagna “Direzione Generale Cura della Persona, Salute e Welfare, Area ICT e transizione digitale dei servizi al cittadino” and reported according to the RECORD (The REporting of studies Conducted using Observational Routinely-collected health Data) Statement [20]. HDR systems routinely collect information for each patient discharged from public and private hospitals for organizational and economic purposes. The number of hospitalizations, number of admitted children (that is different as children can have more than one admission in the study period), mean length of hospitalization, mean and median age at admission were aggregated per year and per age classes 0–28, 29–90, 91–365 days. The number of admissions with a code for intubation, use of CPAP, oxygen, infusion, and chest X-ray procedures were reported. To meet privacy criteria, data submitted to the principal investigator did not contain any identifiable information on patients or caregivers. Ethical approval was therefore not required, since patients’ data were provided in aggregated and anonymous form directly from the Regional Health Service of Emilia-Romagna.

The Emilia-Romagna region covers an area of 22,446 km^2^ in the northern part Italy. Approximately 4,5 million inhabitants live in this area, and ∼30,000 deliveries occur each year.

For each study year, data regarding resident population were obtained from the National Statistics Institute, ISTAT, (available at the website http://dati.istat.it/Index.aspx?DataSetCode=DCIS_POPRES1#). In order to estimate the number of infants with less than 1 year of age, we considered the resident population on January 1st of the following year.

### Statistical analysis

Results were presented as number of cases and percentage or prevalence (x 100) as far as counts were concerned, while continuous data were presented as weighted mean and weighted standard deviation. Weighted t-test was carried out to compare the mean of aggregated data. Z-test was carried out to test the null hypothesis that the proportion of cases in several groups are the same, followed by pairwise Z-test as post-hoc significance testing between pairs with p-values adjusted using the Bonferroni method. A P-value of less than 0.05 was considered as statistically significant.

All analyses were carried out by R version 4.4.0 (R Core Team (2024), R Foundation for Statistical Computing, Vienna, Austria.

## Results

Of the 43,828 hospital admissions registered from 2018 to 2021 for infants aged less than 1 year of age, 4,396 (10%) had an ICD of bronchiolitis. The number of infants admitted is 4,282. Table 1 summarizes the characteristics of the studied population.

Children under 1 year of age admitted for bronchiolitis in 2018, 2019, 2020 and 2021 were 1,257, 1,291, 702 and 1,046, respectively. Considering the whole estimated annual resident population, children under 1 year hospitalized due to bronchiolitis were 3.9% (1,257/ 32,318), 4.2% (1,291/ 30,914), 2.3% (688/ 29,948), 3.5% (1,046/ 30,065) of infants resident in the Emilia-Romagna Region, respectively.

RSV was the cause of bronchiolitis in 67.5% (875/1,297) of cases in 2018, 65.5% (868/1,325) of cases in 2019, 69.4% (494/712) of cases in 2020 and 82% (871/1,062, *p* < 0.01) of cases in 2021.

**Table 1:** Characteristics of the studied population

Characteristics	Studied population
Study period, years	2018–2021
Admission due to bronchiolitis, n	4,396
Admission due to RSV related bronchiolitis, n (%)	3,108 (71)
Children admitted due to bronchiolitis, n	4,282
Mean age on admission, days	99.4 ± 64.9
Age classes, n of admission (%) - 0–28 days - 29–90 days - 91–365 days	694 (16) 1899 (43) 1803 (41)

RSV: respiratory syncytial virus

### Rates of hospital admissions due to bronchiolitis or other causes

**Fig. 1:**
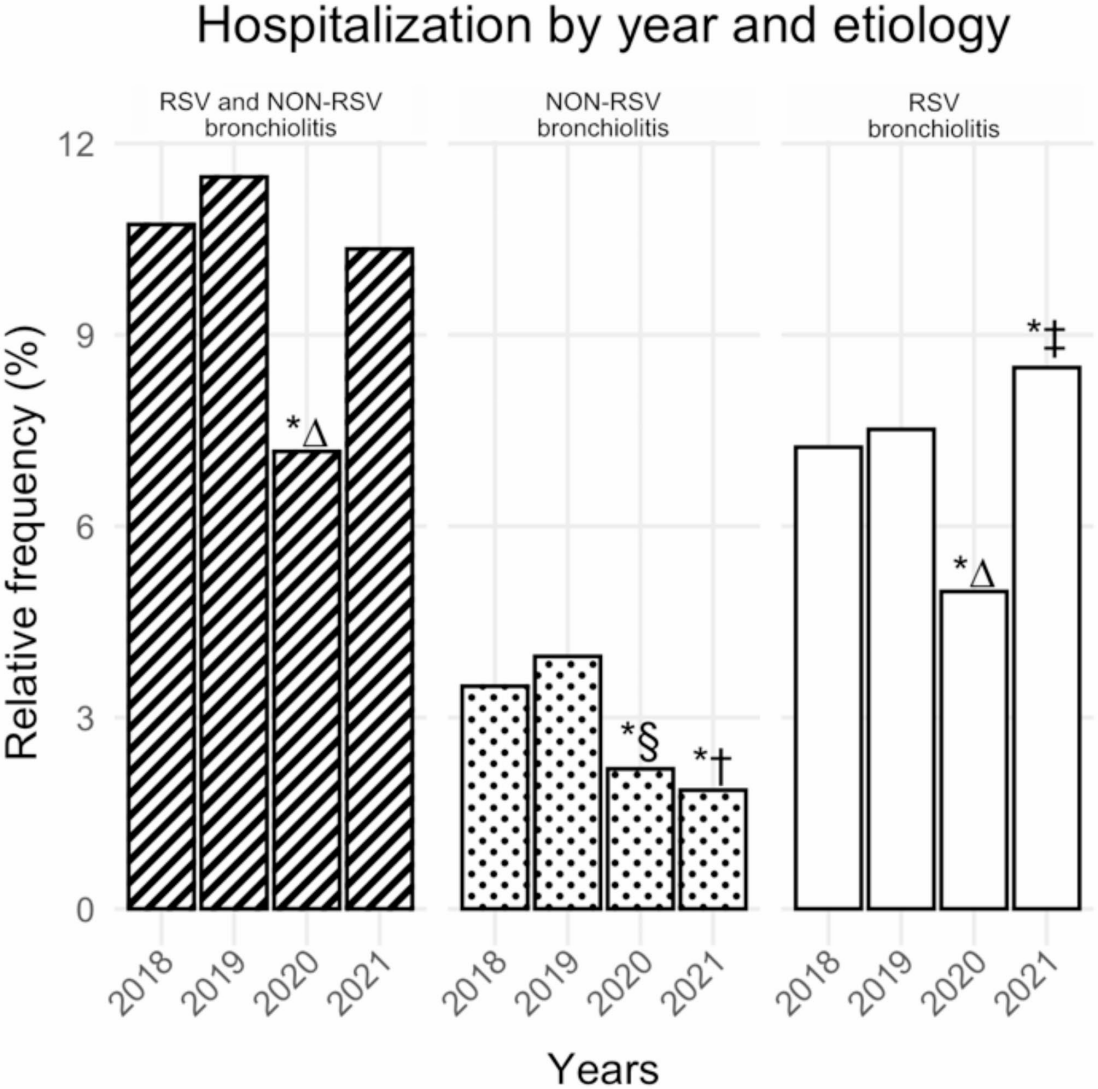
Rates of hospital admission due to bronchiolitis. The y-axis shows the proportion of hospital admissions due to bronchiolitis compared to hospital admissions due to all causes during the first year of life Z-test was carried out to test the null hypothesis that the proportion of cases in several groups are the same, followed by pairwise Z-test as post-hoc significance testing between pairs with p-values adjusted using the Bonferroni method. *p < 0.01, (∆)2020 vs. all other years, (§) 2020 vs. 2018 and 2019, (†) 2021 vs. 2018 and 2019, (‡) 2021 vs. 2018

As compared to all causes, rates of hospital admissions due to bronchiolitis (RSV and non-RSV) declined from 11.5% (1,325/ 11,546) in 2019 to 7.2% (712/9,929 in 2020) (*p* < 0.01), but admissions for RSV bronchiolitis resurged during 2021 (Fig. 1).

### Age on admission

Mean age on admission was 99.4 ± 64.9 days and did not differ between RSV and non-RSV cases (RSV-cases 94.4 ± 63.4 days vs. non-RSV cases 111.6 ± 66.9 days, *p* = 0.54). Noticeably, 59% of children admitted to hospital due to RSV and non-RSV bronchiolitis were aged less than 90 days.

**Fig. 2:**
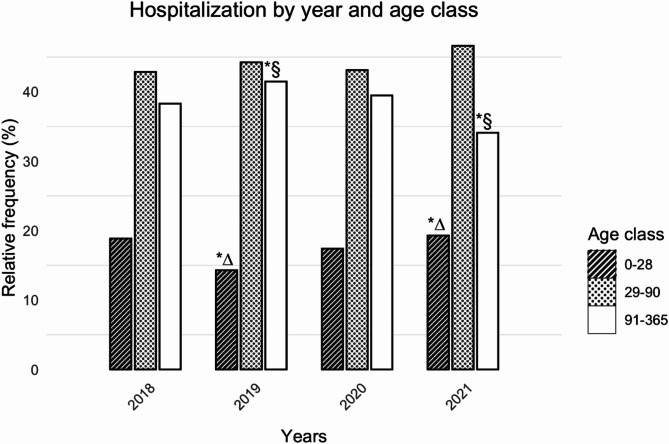
Rates of hospital admission (from 2018 to 2021) due to RSV bronchiolitis according to infants’ age Z-test was carried out to test the null hypothesis that the proportion of cases in several groups are the same, followed by pairwise Z-test as post-hoc significance testing between pairs with p-values adjusted using the Bonferroni method *(∆) Significant differences (2019 vs. 2021, *p* = 0.04) for infants aged 0 to 28 days, *(§) Significant differences (2019 vs. 2021, *p* = 0.01) for infants aged 91 to 365 days

Amongst infants aged 0 to 28 days (Fig. 2), admissions due to RSV bronchiolitis increased from 14.3% (124/868) in 2019 to 19.3% (168/871, *p* = 0.04) in 2021, but declined during the same period (from 41.5%, 360/868 in 2019 to 34.1% 297/871 in 2021, *p* = 0.01) amongst infants aged 91 to 365 days. No differences were found in cases aged 29 to 90 days of age.

### Length of stay, respiratory support and i.v. (intravenous) infusions in RSV and non-RSV bronchiolitis

Mean length of stay was 5.4 ± 0.83 days. Length of stay did not differ between RSV (5.6 ± 0.84 days) and non-RSV bronchiolitis (5 ± 0.62 days, *p* = 0.07).

Only a few infants admitted to hospital (34/4,396, 0.8%) underwent mechanical ventilation, whereas 7.8% (344/4,396 cases) received non-invasive ventilation (NIV); furthermore, 65.7% (2,889/4,396) received oxygen supplementation, 15.0% (659/4,396) underwent at least one chest -X ray and 24.7% (1,084/4,396) received i.v. infusions (i.e., glucosaline or parenteral nutrition). Differences between RSV cases and other cases are presented in Table 2: RSV-related cases were more likely to undergo NIV and oxygen supplementation and to receive infusion.

**Table 2:** Days of hospital stay, oxygen support and non-invasive ventilation; numbers of chest X rays and i.v. infusions

Admissions, *n* (%)	RSV 3108 (70.7)	Non-RSV 1288 (29.3)	RSV and Non-RSV 4396 (100)	*P*-value
Length of stay, mean (SD)	5.6 (± 0.8)	5 (± 0.6)	5.4 (± 0.8)	0.07
Oxygen support, n (%)	2,169 (69.8)	720 (55.9)	2,889 (65.7)	< 0.01
Non-invasive ventilation, n (%)	266 (8.6)	78 (6.1)	344 (7.8)	< 0.01
Chest X ray, n (%)	475 (15.3)	184 (14.3)	659 (15.0)	0.43
I.v. infusions, n (%)	810 (26.1)	274 (21.3)	1,084 (24.7)	< 0.01

RSV: respiratory syncytial virus

n, number of cases

SD: standard deviation

i.v.: intravenous

Z-test was carried out to test the null hypothesis that the proportion of cases in several groups are the same. T-test was carried out to test the null hypothesis that the means in several groups are the same

**Fig. 3:**
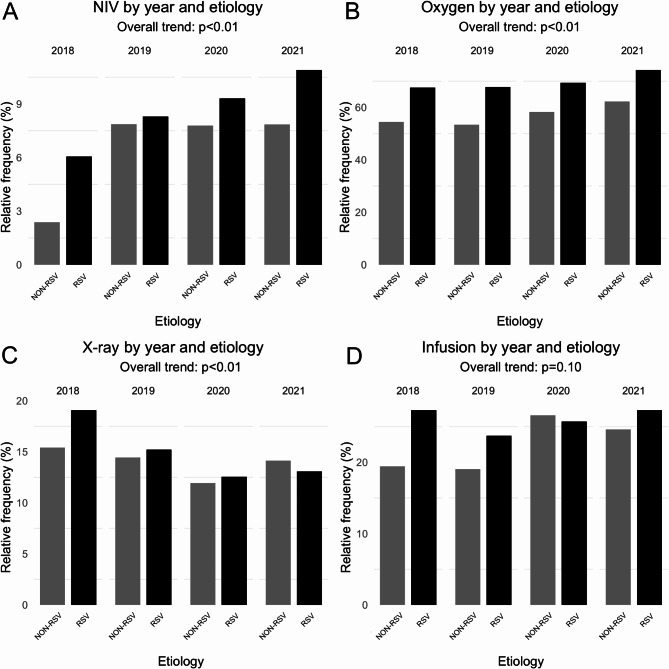
Respiratory support, chest X ray and i.v. infusions during study period (2018–2021) according to RSV and non-RSV bronchiolitis RSV: respiratory syncytial virus NIV: non-invasive ventilation X-ray: chest X ray Chi-squared test for trend was carried out to test for trend in group proportions over years

Considering all cases (Fig. 3), we found an overall significant increase in the use of NIV and oxygen supplementation (*p* < 0.01), and a decrease in chest-X rays (*p* < 0.01). No changes were found regarding i.v. infusions (*p* = 0.1).

## Discussion

The aim of this study was to analyze the epidemiology and characteristics of bronchiolitis in children under one year of age who required hospital admission between 2018 and 2021 in Emilia-Romagna, a region in Northern Italy, and to examine the influence of the COVID-19 pandemic on admissions. We collected region-wide data from all hospitals utilizing aggregate data from Hospital Discharge Records (HDRs) provided by the Regional Health Service.

The analysis was limited to children under one year of age as bronchiolitis is one of the leading causes of hospital admissions in this age group [21–23]. It is well known that infants between 0 and 365 days are most susceptible to severe bronchiolitis complications, often resulting in a significant need for ICU admissions [6, 24]. This, in turn, leads to higher costs for the National Healthcare System [6], therefore, we believe it is vital to have region-based data to effectively implement any prevention campaigns and improve the clinical management of this condition.

During the study period, bronchiolitis accounted for 10% of all admissions in infants under age 1 year, comparable with the 7–13% reported by others [25]. Consistent with previous reports [24, 26, 27], we estimated that in Emilia-Romagna approximately from 2 to 4% of children in their first year of age were hospitalized due to bronchiolitis.

The significant decrease of bronchiolitis-related admissions from 11.5% in 2019 to 7.2% in 2020 was most likely due to the SARS-CoV-2 pandemic and associated preventive measures [3]. As highlighted by others authors, after an initial decrease in bronchiolitis admissions, a resurgence occurred in 2021, with an important change in viral seasonality [28–32]. Unfortunately, we lack data regarding month of admission to clearly document seasonal peaks, but the significant increase in rates of RSV bronchiolitis during 2021 (up to 82% of cases) supports this epidemiological hypothesis [28, 33]. This trend has been also confirmed in previous Italian studies [14, 28, 29].

As bronchiolitis admissions are related to geographical and socioeconomic factors [26], we compared our data mainly with other recent Italian studies regarding bronchiolitis under age 1 year, although such comparisons are problematic due to different settings, samples and aims of the study population.

Consistent with previous reports [14, 34], in the current study most admissions regarded children aged 0–3 months, a highest-risk age group for complications [1]. We found a significant difference in the 0–28 day age group for RSV cases, which accounted for 14.4% of admissions in 2019 and 19.3% in 2021. These increases, consistent with other authors’ findings [28], could be attributed to a reduction in the level of maternal immunoglobulin protection due to decreased virus circulation during the last trimester of pregnancy. Maternal immunoglobulins are known to be protective for the newborn [35].

The mean age on admission for bronchiolitis was 99 days and a mean length of stay was 5.4 days, consistent with previous Italian studies [29, 36, 37].

Several studies report that the hospitalization burden due to bronchiolitis is higher in RSV infected infants [19, 25, 33, 38]. In this study, the severity of RSV cases was supported by an increase rate of respiratory support (use of oxygen and non-invasive ventilation) and the use of i.v. infusion, while we were unable to observe a significant difference in length of hospital stay, age at admission and use of chest X rays between RSV and non-RSV cases.

The overall (RSV and non-RSV cases) significant increasing trend in the use of oxygen and non-invasive ventilation could either be a consequence of an increase in the percentage of RSV infection, particularly in the last analyzed year, or a change in clinical habits [39]. Indeed, we found a statistically significant trend in the reduction of chest X-ray usage. This finding can be interpreted as a result of better adherence to national guidelines on bronchiolitis management, as reported in other national studies [36].

## Strengths and limitations

The strength of our study is its area-based design, with a homogeneous set of data, encompassing a period prior to and after the SARS-CoV-2 pandemic. As already discussed, there are several valuable Italian studies, but their data comes from single centers or hospital networks, whereas to our knowledge only one area-based study on bronchiolitis has been published to date in Italy, and it addresses only infections prior to the SARS-CoV-2 pandemic [19].

The main limitation of this study is the retrospective designs as well as the information coming only from HDR and including only aggregated data.

HDRs, which are collected mainly for organizational and economic purposes, present several limitations and enable a low level of clinical detail to be obtained. For example, the ICD9-CM coding system does not have a specific code for high flow nasal cannula which has only been used in paediatric clinical practice in recent years; thus, the coding of this specific respiratory support is likely to be heterogeneous. Month of admission and gender were also unknown.

Therefore, HDRs have proven to be a simple and useful tool, although they require to be combined with additional clinical data to obtain more complete information.

## Conclusions

We observed a reduction in bronchiolitis-related hospitalizations due to COVID-19 restrictions, with a resurgence of RSV infections after measures were reduced. We also provide data regarding hospital treatment and length of stay, with increased rates of respiratory support and i.v. infusions after RSV bronchiolitis.

Our data would be useful in establishing the impact of future universal prevention measures.

## Data Availability

The datasets generated during and/or analyzed during the current study are not publicly available as they have been entrusted to the authors by the Regional Health Service of Emilia-Romagna, but are available from the corresponding author on reasonable request. Acknowledgments: We would like to acknowledge the Regional Health Service of Emilia-Romagna “Direzione Generale Cura della Persona, Salute e Welfare, Area ICT e transizione digitale dei servizi al cittadino”.

## List of abbreviations

CPAP: Continuous positive airway pressure
EMA: European Medicines Agency.
FDA: Food and Drug Administration
HDR: Hospital discharge records
ICD9-CM: International Classification of Disease, 9th Revision, Clinical Modification
ISTAT: National Statistics Institute
LRTI: Lower respiratory tract infection
NIV: Non-invasive ventilation
RSV: Respiratory syncytial virus
X ray: Chest -X ray

## References

[1] Manti S, Staiano A, Orfeo L, Midulla F, Marseglia GL, Ghizzi C, et al. UPDATE– 2022 Italian guidelines on the management of bronchiolitis in infants. Ital J Pediatr. 2023;NLM Medline:19. 10.1186/s13052-022-01392-6 PMC9912214 36765418

[2] Dalziel SR, Haskell L, O’Brien S, Borland ML, Plint AC, Babl FE, et al. Bronchiolitis. The Lancet. Elsevier B.V.; 2022. pp. 392–406. 10.1016/S0140-6736(22)01016-9 35785792

[3] Azzari C, Baraldi E, Bonanni P, Bozzola E, Coscia A, Lanari M et al. Epidemiology and prevention of respiratory syncytial virus infections in children in Italy. Ital J Pediatr. BioMed Central Ltd; 2021. 10.1186/s13052-021-01148-8 PMC8487331 34600591

[4] Shi T, McAllister DA, O’Brien KL, Simoes EAF, Madhi SA, Gessner BD, et al. Global, regional, and national disease burden estimates of acute lower respiratory infections due to respiratory syncytial virus in young children in 2015: a systematic review and modelling study. Lancet. 2017;390:946–58. 28689664 10.1016/S0140-6736(17)30938-8 PMC5592248

[5] Geoghegan S, Erviti A, Caballero MT, Vallone F, Zanone SM, Losada JV, et al. Mortality due to respiratory syncytial virus burden and risk factors. Am J Respir Crit Care Med. 2017;195:96–103. 27331632 10.1164/rccm.201603-0658OC

[6] Dovizio M, Veronesi C, Bartolini F, Cavaliere A, Grego S, Pagliaro R et al. Clinical and economic burden of respiratory syncytial virus in children aged 0–5 years in Italy. Ital J Pediatr [Internet]. 2024;50:57. Available from: https://ijponline.biomedcentral.com/articles/10.1186/s13052-024-01628-7 10.1186/s13052-024-01628-7 PMC10964524 38528616

[7] Wildenbeest JG, Billard MN, Zuurbier RP, Korsten K, Langedijk AC, van de Ven PM, et al. The burden of respiratory syncytial virus in healthy term-born infants in Europe: a prospective birth cohort study. Lancet Respir Med. 2023;11:341–53. 36372082 10.1016/S2213-2600(22)00414-3 PMC9764871

[8] Young M, Smitherman L. Socioeconomic impact of RSV hospitalization. Infect Dis Ther. Adis; 2021. pp. 35–45. 10.1007/s40121-020-00390-7 PMC7926081 33656651

[9] Martinón-Torres F, Navarro-Alonso JA, Garcés-Sánchez M, Soriano-Arandes A. The path towards effective respiratory Syncytial Virus immunization policies: recommended actions. Arch Bronconeumol. Sociedad Espanola de Neumologia y Cirugia Toracica (SEPAR); 2023. pp. 581–8. 10.1016/j.arbres.2023.06.006 37414639

[10] Ghirardo S, Ullmann N, Ciofi degli Atti ML, Raponi M, Cutrera R. Delayed season’s onset and reduction of incidence of bronchiolitis during COVID-19 pandemic. Pediatr Pulmonol. John Wiley and Sons Inc; 2021. pp. 2780–1. 10.1002/ppul.25461 PMC8242365 34003595

[11] Loconsole D, Centrone F, Rizzo C, Caselli D, Orlandi A, Cardinale F et al. Out-of-season epidemic of respiratory Syncytial Virus during the COVID-19 pandemic: the high burden of child hospitalization in an Academic Hospital in Southern Italy in 2021. Children. 2022;9. 10.3390/children9060848 PMC9221938 35740785

[12] Maison N, Peck A, Illi S, Meyer-Buehn M, von Mutius E, Hübner J, et al. The rising of old foes: impact of lockdown periods on non-SARS-CoV-2 viral respiratory and gastrointestinal infections. Infection. 2022;50:519–24. 35076891 10.1007/s15010-022-01756-4 PMC8787179

[13] Foley DA, Yeoh DK, Minney-Smith CA, Martin AC, Mace AO, Sikazwe CT, et al. The Interseasonal resurgence of respiratory Syncytial Virus in Australian Children following the reduction of Coronavirus Disease 2019-Related Public Health measures. Clinical infectious diseases. Oxford University Press; 2021. pp. E2829–30. 10.1093/cid/ciaa1906 PMC7929151 33594407

[14] Lodi L, Catamerò F, Voarino M, Barbati F, Moriondo M, Nieddu F et al. Epidemiology of respiratory syncytial virus in hospitalized children over a 9-year period and preventive strategy impact. Front Pharmacol. 2024;15. 10.3389/fphar.2024.1381107 PMC11150665 38841370

[15] Zheng Z, Pitzer VE, Shapiro ED, Bont LJ, Weinberger DM. Estimation of the timing and intensity of reemergence of respiratory Syncytial Virus following the COVID-19 pandemic in the US. JAMA Netw Open. 2021;4. 10.1001/jamanetworkopen.2021.41779 PMC8678706 34913973

[16] Bechini A, Salvati C, Bonito B, Del Riccio M, Stancanelli E, Bruschi M et al. Costs and healthcare utilisation due to respiratory syncytial virus disease in paediatric patients in Italy: a systematic review. Public Health. Elsevier B.V.; 2024. pp. 103–11. 10.1016/j.puhe.2023.11.039 38154422

[17] Villani A, Vittucci AC, Antilici L, Pisani M, Scutari R, Di Maio VC et al. Prevention of RSV Bronchiolitis: An Ethical Issue. Pediatric Infectious Disease Journal [Internet]. 2024; Available from: https://journals.lww.com/10.1097/INF.0000000000004354 10.1097/INF.0000000000004354 38621157

[18] European Medicines Agency. https://www.ema.europa.eu/en/medicines/human/EPAR/beyfortus

[19] Cutrera R, d’Angela D, Orso M, Guadagni L, Vittucci AC, Bertoldi I et al. Trends in hospitalizations of children with respiratory syncytial virus aged less than 1 year in Italy, from 2015 to 2019. Ital J Pediatr. 2024;50. 10.1186/s13052-024-01688-9 PMC11191168 38902751

[20] Benchimol EI, Smeeth L, Guttmann A, Harron K, Moher D, Peteresen I et al. The REporting of studies conducted using Observational routinely-collected health data (RECORD) Statement. PLoS Med. 2015;12. 10.1371/journal.pmed.1001885 PMC4595218 26440803

[21] Ehlken B, Ihorst G, Lippert B, Rohwedder A, Petersen G, Schumacher M, et al. Economic impact of community-acquired and nosocomial lower respiratory tract infections in young children in Germany. Eur J Pediatr. 2005;164:607–15. 15965766 10.1007/s00431-005-1705-0

[22] Zepeda TJ, Vásquez ZJ, Delpiano ML. Costos directos de infección respiratoria baja por VRS en menores de un año. Rev Chil Pediatr. 2018;0–0. 10.4067/S0370-41062018005000401 30571819

[23] Gastaldi A, Donà D, Barbieri E, Giaquinto C, Bont LJ, Baraldi E. Covid-19 lesson for respiratory syncytial virus (rsv): Hygiene works. Children. MDPI; 2021. 10.3390/children8121144 PMC8700687 34943339

[24] Cocchio S, Prandi GM, Furlan P, Venturato G, Saia M, Marcon T et al. Respiratory Syncytial Virus in Veneto Region: Analysis of Hospital Discharge Records from 2007 to 2021. Int J Environ Res Public Health. 2023;20. 10.3390/ijerph20054565 PMC10002215 36901576

[25] Faraguna MC, Lepri I, Clavenna A, Bonati M, Vimercati C, Sala D et al. The bronchiolitis epidemic in 2021–2022 during the SARS-CoV-2 pandemic: experience of a third level centre in Northern Italy. Ital J Pediatr. 2023;49. 10.1186/s13052-023-01425-8 PMC9942300 36803828

[26] Noble M, Khan RA, Walker B, Bennett E, Gent N. Respiratory syncytial virus-associated hospitalisation in children aged ≤ 5 years: a scoping review of literature from 2009 to 2021. ERJ Open Res. 2022;8. 10.1183/23120541.00593-2021 PMC9149382 35651366

[27] Martinón-Torres F, Carmo M, Platero L, Drago G, López-Belmonte J, Bangert M et al. Clinical and economic hospital burden of acute respiratory infection (BARI) due to respiratory syncytial virus in Spanish children, 2015–2018. BMC Infect Dis. 2023;23. 10.1186/s12879-023-08358-x PMC10249572 37291530

[28] Parola F, Brach Del Prever A, Deut V, Costagliola G, Guidi C, Ragusa N et al. Impact of SARS-CoV-2 Pandemic and Lockdown on the HRSV Circulation: Experience of Three Spoke Hospitals in Northern Italy. 2024; Available from: https://www.mdpi.com/journal/viruses 10.3390/v16020230 PMC10891764 38400006

[29] Ghirardo S, Ullmann N, Zago A, Ghezzi M, Minute M, Madini B et al. Increased bronchiolitis burden and severity after the pandemic: a national multicentric study. Ital J Pediatr. 2024;50. 10.1186/s13052-024-01602-3 PMC10865582 38350986

[30] Eden JS, Sikazwe C, Xie R, Deng YM, Sullivan SG, Michie A et al. Off-season RSV epidemics in Australia after easing of COVID-19 restrictions. Nat Commun. 2022;13. 10.1038/s41467-022-30485-3 PMC9130497 35610217

[31] Guitart C, Bobillo-Perez S, Alejandre C, Armero G, Launes C, Cambra FJ et al. Bronchiolitis, epidemiological changes during the SARS-CoV-2 pandemic. BMC Infect Dis. 2022;22. 10.1186/s12879-022-07041-x PMC8785150 35073855

[32] La Ramos B, Saloni-Gomez N, Ilundain López de Munain A, Fernández-Montero A, Viguria N, López Fernández L, et al. The long-term boomerang effect of COVID-19 on admissions for non-COVID diseases: the ECIEN-2022 study. Eur J Pediatr. 2023;182:4227–36. 37452843 10.1007/s00431-023-05101-1

[33] Boccard V, Prevost B, Denamur S, Peulier-Maitre E, Nathan N, Corvol H. Bronchiolitis: increased severity in the post-COVID-19 era. Pediatr Pulmonol. 2024. 10.1002/ppul.27172 PMC11601030 38990099

[34] Baldassarre ME, Loconsole D, Centrone F, Caselli D, Martire B, Quartulli L et al. Hospitalization for bronchiolitis in children aged ≤ 1year, Southern Italy, year 2021: need for new preventive strategies? Ital J Pediatr. 2023;49. 10.1186/s13052-023-01455-2 PMC10243233 37280662

[35] Koivisto K, Nieminen T, Mejias A, Capella Gonzalez C, Ye F, Mertz S, et al. Respiratory syncytial virus (RSV)-Specific antibodies in pregnant women and subsequent risk of RSV hospitalization in Young infants. J Infect Dis. 2022;225:1189–96. 34129040 10.1093/infdis/jiab315 PMC8974854

[36] Abbate F, Depietri G, Tinelli C, Massimetti G, Picariello S, Peroni D, et al. Impact of the publication of the Italian guidelines for bronchiolitis on the management of hospitalized children in Pisa, Italy. Pediatr Pulmonol. 2023;58:2267–74. 37154513 10.1002/ppul.26460

[37] Bozzola E, Ciarlitto C, Guolo S, Brusco C, Cerone G, Antilici L et al. Respiratory Syncytial Virus Bronchiolitis in Infancy: the Acute hospitalization cost. Front Pediatr. 2021;8. 10.3389/fped.2020.594898 PMC7848214 33537260

[38] Rodriguez-Fernandez R, González-Sánchez MI, Perez-Moreno J, González-Martínez F, de la Mata Navazo S, Mejias A, et al. Age and respiratory syncytial virus etiology in bronchiolitis clinical outcomes. J Allergy Clin Immunology: Global. 2022;1:91–8. 10.1016/j.jacig.2022.05.005 PMC10509905 37781264

[39] Ghirardo S, Cozzi G, Tonin G, Risso FM, Dotta L, Zago A, et al. Increased use of high-flow nasal cannulas after the pandemic in bronchiolitis: a more severe disease or a changed physician’s attitude? Eur J Pediatr. 2022;181:3931–6. 36083314 10.1007/s00431-022-04601-w PMC9458479

